# Lessons from the Bubonic Plague

**Published:** 1914-08

**Authors:** 


LESSONS FROM THE BUBONIC PLAGUE.—Every threatened epidemic results in good. It is a law of nature that nothing is so bad but that some good comes from it. New Orleans has undergone a scare that will be of great benefit to all the coast States. A hard war is being made on rats and their boon companions—the fleas. It is said that the fleas that infest certain kinds of rats, and not the rats, are the bearers of the bubonic plague. But the rat must be exterminated in order to kill the flea.

Dr. Rupert Blue, Surgeon General of the Public Health Department, says that, while the catching of rats is all right so far as it goes, the permanent fight against the importation of disease-bearing rats must be made by the erection of concrete structures along the water fronts. In one concrete pier at Galveston only one rat was found, while an old wooden pier was literally filled with rats of all kinds, sizes and breeds.

Of course, following this scare and Dr. Blue's report, the old wooden wharves and other wood structures along the Galveston water front will be replaced by concrete structures, as they should be. The result will be a far more sightly water front, and a more healthy city. The city authorities are alert to the importance of the movement to exterminate rats. Dr. Blue, in an interview, said:

"I am gratified at the spirit of desire on the part of your city health officers to make the city free from the pest, and would urge that the city officials and citizenship of the island back them up with the necessary funds and co-operation. Certainly one of the first steps in this direction should be an ordinance regulating building along the water front. San Francisco, Oakland and Seattle have such ordinances today, and these cities are virtually free from the rat and the plague. New Orleans is planning to adopt such an ordinance at once."

This movement should extend to every other coast point in the country, and the work of extermination should extend throughout the country. The economic waste in this country from rats can hardly be estimated. It is said that rats cost the country at least one cent a day each for their maintenance. Almost every farm in the country, and nearly every city and town home, is supporting many of the dead-beat boarders at an enormous total expense to the country. The fight should be kept up until the country is clear of rats and mice. This can be accomplished in a short while by concerted action, and now, that the coast cities are aroused, the immigrant rat will soon be unable to find a "welcome" at any of our seaports.

It is said that all rats do not carry the plague-scattering flea about their clothing, but all rats do help themselves to whatever their appetites may fancy.

Dr. Blue stated that there are four varieties of the rat family which are spreaders of the plague—the family names being Mus. There is first of all the Mus norvegicus, which lives in sewers and in the low, dark places and is a brownish gray. He is a very ferocious animal and makes it impossible for rats of a less vigorous frame and vitality to live where they are around.

Next comes the Mus rattus, which is a black rat or India rat, which lives on second or upper floors, and is spoken of as the 'porch climber'; thirdly, there is the Mus Alexandrinus, which also lives in the upper floors; while the fourth variety is the Mus muscusvus, or the mouse, which is the least dangerous of the four. To the first three we may attribute all cases of the bubonic plague.

On the Galveston wharves some 300 rats are being caught every day. The official rat-cather says that the best way to set a trap is to have it covered with a box or sacking, for rats will not touch a wire trap unless exceedingly hungry. The best way is to have a box completely covering the trap, with but a small door in it that leads directly into the door of the trap. In this way many rats are enticed which otherwise would not approach the traps.

For bait the best thing is watermelon seeds. At the present time he is using watermelon seeds, with a small square of the melon itself in each trap, and he says the rodents are very eager to get at this delicacy. Another good bait, he states, is bread and salmon, but this is not so effective here because much of it is carried off by the roaches that infest the water front. Any sort of bait that will attract the large rats will do, however, but those mentioned will produce the best results.

In the interest of public health, as well as for economical reasons, physicians everywhere should co-operate with the public health departments in the effort to exterminate rats, and where there is no public sentiment against the rodents it should be created by local physicians in conjunction with the health authorities.

The *Houston Post's* statistician has been making some figures on the cost of keeping rats in Houston, and makes the following apt editorial comment:

"Dr. Rupert Blue, Surgeon General of the United States Army, spent Tuesday in Houston in connection with the precautions that he is taking to prevent the spread of the bubonic plague from New Orleans—the only present known source of infection on the American continent. He urges a vigorous campaign for the destruction of our rat population, which he estimates approximates 300,000, or about three rats to every human being. To feed these rats, he says, costs us $45,000 a month, or $540,000 a year. That is a pretty hefty sum of money to be wasted in sustaining such an undesirable population. Furthermore, Dr. Blue says that the bubonic plague is never transmitted from person to person, but always from rat to rat or rat to person. Rats, he says, as a usual thing have from 150 to 200 fleas on them, and the flea is therefore what carries the disease to human beings. If, as Dr. Blue estimates, there are 300,000 rats in Houston, there are just that many rats that ought not to exist here. We don't need them—especially when we have to pay $1500 a day for their upkeep. A 10-cent bounty for each rat would cost the city only $30,000 for the extermination of the estimated 300,000. The bounty would be a powerful incentive to rat extermination; also, it appeals to the *Post* as a good investment."

